# Editorial: Dietary factors, epigenetics and their implications for human obesity - volume II

**DOI:** 10.3389/fendo.2023.1328944

**Published:** 2023-12-11

**Authors:** Omar Ramos-Lopez

**Affiliations:** Medicine and Psychology School, Autonomous University of Baja California, Tijuana, Baja California, Mexico

**Keywords:** epigenetics, obesity, diet, nutrients, DNA methylation, gene expression, histones, miRNA

The obesity epidemic remains a major health concern worldwide due to rising prevalence rates and the negative impact on people´s lives and well-being. In fact, obesity is considered a risk factor for the onset and progression of a number of comorbidities including metabolic diseases such as type 2 diabetes mellitus, cardiovascular events, hypertension, fatty liver disease, and some cancers. The physiopathological processes underlying these diseases include insulin resistance, dyslipidemia, hyperinflammation, dysbiosis of the gut microbiota and dysregulation of hormones and peptides.

Several factors are involved in the development of excessive body adiposity. Long-term consumption of an unbalanced, energy-dense diet rich in saturated fats and sugars, combined with a lack of essential nutrients has been identified as one of the main environmental factors contributing to the development of obesity. In addition, epigenetic signatures (i.e. deoxyribonucleic acid methylation [DNAm], long non-coding ribonucleic acids [lncRNAs], and histone dynamics) may influence susceptibility to obesity via changes in the expression of key metabolic genes. Indeed, there is growing evidence that one of the mechanisms by which nutrients can influence metabolism is through epigenetic signatures (1). The nutritional status of the mother during periconception is also critical for fetal growth and future offspring health, with epigenetic processes playing a role. Moreover, close interactions between telomere length and epigenetic status can influence the phenotype of mammalian cells (2).

The present Research Topic includes five original articles and two reviews that provides new insights into obesity research by analyzing the interplay between nutrition and epigenetic phenomena. Rapps et al. investigated the role of lysine demethylase 4D (kdm4d), a specific demethylase of residue histone H3 lysine 9 (H3K9), in energy homeostasis via modulation of the expression of agouti-related protein (AgRP), an important neuropeptide that regulates hunger/satiety responses in a rodent model of diet-induced obesity (DIO). They found a downregulation of *Kdm4d* in DIO, leading to an enrichment of H3K9 dimethylation (H3K9me2) at the *AgRP* promoter and consequently to a transcriptional repression of AgRP, which was reversed by calorie restriction. They also demonstrated that *in vivo* inhibition of kdm4d activity by the selective pharmacological agent JIB-04 (a pan Jumonji histone demethylase inhibitor) induced transcriptional repression of AgRP, therefore reducing food signaling. They propose that demethylation of H3K9 by kdm4d is critical for maintaining a stable epigenetic shape of the AgRP promoter and thus represents a potential target for the treatment of obesity.

Environmental factors such as eating behavior or physical activity may interact with the epigenome and influence the development of obesity. More importantly, clinical trials have documented changes in epigenetic marks associated with obesity during lifestyle interventions to promote weight loss. In this Research Topic, Aurich et al. provide an overview of recent human intervention studies that have used candidate-based and genome-wide DNAm approaches (with particular emphasis on methylation age and *in-utero* assays) to investigate the epigenetic landscape underlying lifestyle-mediated weight loss. This review highlights the potential of targeted lifestyle prescriptions to modify obesity-related epigenetic patterns (mainly DNAm), thereby gaining a better understanding of epigenetic reprogramming in obesity. However, the authors recommend further robust and larger studies to identify additional specific DNAm biomarkers that modulate obesity outcomes.

Endocrine disruptors are a group of widely used natural or synthetic molecules that can interfere with normal endocrine signalling pathways, leading to adverse health outcomes, including hormonal disruption associated with obesity. Of note, exposure to these compounds is usually due to the consumption of foods that can accumulate in key endocrine tissues (e.g. adipose tissue) and affect the epigenome. In this context, Brennan et al. investigated whether polychlorinated biphenyls (PCBs) are associated with circulating micro ribonucleic acids (miRNAs) and hormonal characteristics in non-obese women with polycystic ovary syndrome (PCOS), with comparable healthy women serving as a control group. They found significant correlations between certain miRNAs and PCBs (i.e. miR-26a-5p, miR-193a-5p, miR-2110, miR-195-5p, miR-99b-5p, miR-146b-5p, miR-139-5p and miR-146b-5p), and some of them were simultaneously associated with menstrual cycle factors in healthy controls. They conclude that the effects of PCBs on miRNAs may lead to changes in the hormones of the hypothalamic-ovarian axis, which may thus affect fertility.

Nutritional imbalances during pregnancy can predispose to the development of further diseases such as obesity and diabetes in the offspring. Epigenetic changes are one of the mechanisms by which exposure to an altered intrauterine environment can influence the health status of offspring later in life. Rodolaki et al. provide a review of the current literature examining the effects of maternal diabetes on the future health and neurological development of offspring. Experimental and clinical data have linked maternal diabetes to neurological damage such as motor and cognitive impairments, autism spectrum disorders, attention deficit hyperactivity syndrome, learning disabilities, and psychiatric conditions, which may be related to various processes such as epigenetic aberrations, neuroinflammation, iron deficiency, dyslipidemia, and structural brain abnormalities. They conclude that monitoring hyperglycemia during pregnancy should be optimally controlled to mitigate the negative impact of maternal diabetes on offspring neurodevelopment.

In this context, Martins et al. investigated the effects of streptozotocin (STZ)-induced maternal glucose intolerance in rats on the metabolic status of the offspring during pregnancy and in early adulthood. They found that the offspring of STZ-treated mothers (especially in combination with a high-fat diet after weaning) were more prone to develop metabolic impairments in adulthood, which was associated with altered hypothalamic expression of proopiomelanocortin, mainly in females. The authors conclude that maternal dysglycemia can disrupt hypothalamic circuits regulating energy homeostasis, with female offspring being more severely affected.

Epigenetic phenomena regulate telomere length and related enzyme activity, which influence telomere structure and maintenance. Telomere status is involved in the pathobiology of several human diseases, including obesity, and is controlled by several epigenetic influences. Wang et al. analyzed the relationship between body weight range and telomere length in a retrospective cohort and found that weight range was inversely associated with telomere length in American adults. The authors point out that greater weight fluctuations may accelerate telomere shortening and aging, supporting the theory of maintaining a stable normal weight and emphasizing the importance of assessing weight range in the clinical setting. Furthermore, Han et al. explored the effects of telomere length on the risk of adverse pregnancy outcomes using a Mendelian randomization approach using data from genome-wide association studies in European participants. The results of this study provided robust evidence of an association between shorter telomeres and an increased risk of spontaneous abortion. As dietary factors are known to influence telomere length, these results could facilitate the design on dietary strategies to promote health and reduce disease risk by modulating telomeres.

Overall, the original and review articles in this Research Topic contribute important insights into the links between diet, epigenetics, and human obesity. They improve the current understanding of the role of epigenetic backgrounds in energy balance and obesity status and contribute to the development of new nutritional approaches based on epigenetically active dietary components. Future directions in this area include combining epigenetic knowledge with other “omics” tools to provide a more integrative/holistic view of the molecular mechanisms underlying the pathogenesis of obesity. In this regard, the interactions of the epigenome with the gut microbiome, metabolome, genome and proteome should be further investigated.

## Author contributions

OR-L: Conceptualization, Project administration, Writing – original draft, Writing – review & editing.

## References

[1] Milagro FI Mansego ML De Miguel C Martínez JA. Dietary factors, epigenetic modifications and obesity outcomes: progresses and perspectives. Mol Aspects Med (2013) 34(4):782–812. doi: 10.1016/j.mam.2012.06.010 22771541

[2] Benetti R García-Cao M Blasco MA. Telomere length regulates the epigenetic status of mammalian telomeres and subtelomeres. Nat Genet (2007) 39(2):243–50. doi: 10.1038/ng1952 17237781

